# Can Pioglitazone Safeguard Patients of Lichen Planus Against Homocysteine Induced Accelerated Cardiovascular Aging and Reduced Myocardial Performance: A Systematic Review

**DOI:** 10.7759/cureus.12372

**Published:** 2020-12-30

**Authors:** Prachi Balani, Angel R Lopez, Chelsea Mae N Nobleza, Mariah Siddiqui, Parth V Shah, Safeera Khan

**Affiliations:** 1 Internal Medicine, California Institute of Behavioral Neurosciences & Psychology, Fairfield, USA; 2 Psychiatry, California Institute of Behavioral Neurosciences & Psychology, Fairfield, USA; 3 Neurology, California Institute of Behavioral Neurosciences & Psychology, Fairfield, USA; 4 Neurology, St. George's University, True Blue, GRD; 5 Medicine, California Institute of Behavioral Neurosciences & Psychology, Fairfield, USA

**Keywords:** lichen planus, pioglitazone, homoscysteine, arterial aging, arterial stiffness, cardiac remodelling, left ventricle, myocardial performance index, thiazolidinediones, microcirculaiton and inflammation

## Abstract

Lichen planus (L.P.) is a long-standing mucocutaneous inflammatory condition. A less familiar but essential illness association is increased arterial stiffness, endothelial dysfunction, and advanced atherosclerosis. Enhanced cardiac reconditioning and reduced performance of the heart have been suggested. Thiazolidinediones were commenced to manage hyperglycemia in diabetes mellitus. Recently, the class attained popularity after its action on vascular physiology was discovered. With this review, we attempted to explore whether an antidiabetic drug, pioglitazone (PIO), a peroxisome proliferator‑activated receptor γ (PPAR gamma) agonist, can defend patients of lichen planus against increased arterial stiffness and cardiac changes. We methodically screened numerous databases using focused words and phrases for relevant articles. After a comprehensive exploration, we applied the inclusion and exclusion criteria and performed a quality appraisal. Items retained were exhaustively studied. High homocysteine (HHcy) levels in lichen planus play a significant role in modifying the arteries and leading to their dysfunction. Not only does homocysteine affect the precursor cells, but it also increases the free radical damage. Arterial damage and upraised resistance encountered by the heart reduce its performance. After an exhaustive analysis, in our opinion, pioglitazone works in various miscellaneous ways to mitigate the homocysteine mediated changes. Early inclusion of the drug in managing patients with lichen planus seems promising in minimizing the harmful effects of high homocysteine. Evaluating the risk-benefit ratio, we believe that a trial of pioglitazone could be given to patients without underlying cardiac conditions.

## Introduction and background

Lichen ruber planus, or simply lichen planus (L.P.), was first put forward by Dr. Erasmus Wilson in 1869. It is a chronic cell-mediated, autoimmune, inflammatory disease that has an incidence of 0.4-1.9% worldwide. L.P. is the primary representative of lichenoid conditions and is depicted by small papules, often associated with overwhelming itching [1]. The "5P's Disease," as we traditionally remember, affects not only the skin, hair, nails, but also the mucous membranes of the genital tract, gastrointestinal tract, and the eyes [2]. Though the disease's exact etiology is unknown, one of the proposed molecular pathogenesis theories suggests triggering factors that lead to intrinsic or foreign antigens' presentation [1]. Basal keratinocytes are presented as exotic antigens to activated CD8+T cells due to the underlying inflammation. The upregulation of cytokines and intercellular adhesion molecule-1 (I-CAM-1) correlated with the T-helper one cells has also been suggested in this pathogenesis [3]. Studies have shown raised levels of homocysteine and fibrinogen levels in these patients.

Vascular aging is a process that affects all three layers of an artery. At the intima, endothelial dysfunction occurs due to inflammation and reduced vasodilation due to reduced nitric oxide production. The media shows less elastin and an increased amount of collagen, contributing to increased arterial stiffness. Loss of innervation and increased fat deposition at the adventitia further contributes to inflammation [4]. Changes in arterial stiffness and alterations in the mechanical properties are measured by noninvasive markers like pulse pressure (PP), pulse waveform velocity (PWV), and augmentation index (AI) [5,6]. Pulse waves generated during each cardiac contraction travel from the heart until they encounter either resistance or a branch point, where they are then reflected. These reflected waves appear late when the arteries are elastic. However, as the stiffness increases, these waves appear early and with more incredible wave-pulse velocity. A part of the reflection is also sent to the aorta, which results in limited wave energy to the peripheral arteries, thus preventing damage to the microcirculation [7]. Pulse wave velocity is measured as PWV = Length (meters)/ΔTime (seconds). Length is the distance between the two points of the recording, and ΔTime is the time difference between the appearance of a pulse wave at these two points relative to the peak of the R wave on the electrocardiogram. The value of PWV increases with arterial stiffness. The Moens‐Korteweg equation defines factors that affect the amount of PWV, PWV =√(EH/2ρR), E is Young's modulus of the arterial wall, H is the thickness of the wall, R is the radius of the artery at the end of the diastole, and ρ is the density of blood [8]. This tells us that reduced arterial elasticity, diameter, and increased thickness, increase PWV, and damage the microcirculation leading to an increased cardiac workload [7]. Increased arterial aging is thus known to mediate cardiac remodeling and reduce cardiac functions. Two persistent inflammatory diseases of the skin that are frequently credited with an increased risk of thrombosis and cardiovascular disease are L.P. and Psoriasis [9]. Certain studies have shown a significant increase in blood homocysteine levels is associated with the severity of oral lichen planus (O.L.P) [10]. Others illustrate an association with the duration of the disease and not the severity. High homocysteine (HHcy) can lead to the earlier onset of atherosclerotic cardiovascular disease.

Pioglitazone is therapeutically used in patients with diabetes and obesity. However, investigators have not explored pioglitazone as a vasculoprotective drug in Lichen Planus. Also, cardiovascular aging in lichen planus is relatively a topic less looked at and needs more attention. Our review article focuses on homocysteine as the modifiable culprit and its mediated effects on vascular aging and cardiac dysfunction in patients of lichen planus. We further investigate our concern, whether pioglitazone can be used as a potential drug, in the future, in patients with lichen planus to curtail arterial aging and mediated myocardial dysfunction.

## Review

Methods

**Figure 1:**
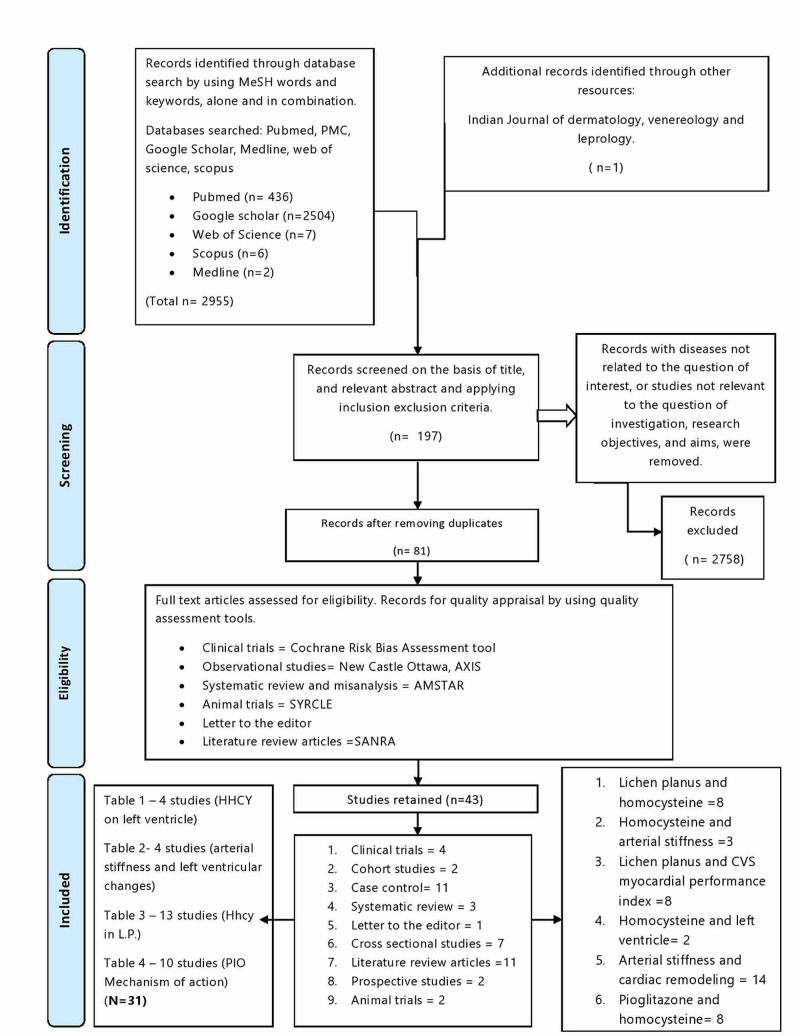
PRISMA Flowchart Showing The Methodology. PRISMA- Preferred Reporting Items for Systematic reviews and Meta-Analysis, PMC- PubMed Central, AXIS- appraisal tool for cross-sectional studies, AMSTAR- a measurement tool to assess systematic reviews, SYRCLE- systematic review center for laboratory animal experimentation, SANRA- scale for the assessment of narrative review articles, L.P.- lichen planus, CVS- cardiovascular system, PIO- pioglitazone

We obeyed the Preferred Reporting Items for Systematic reviews and Meta-Analysis (PRISMA) guidelines for conducting our systematic review. As shown below in Figure 1, we systematically searched multiple electronic databases, such as PubMed, PubMed Central, Web of Science, Google Scholar, Scopus, and Medline for data collection. We explored the database by using terms of medical subject heading (MeSH) and keywords: "lichen planus," "homocysteine," arterial stiffness," "vascular aging," "myocardial performance index," "left ventricle, "and "pioglitazone," separately and in combination to find relevant studies. We performed a nonautomated search on the reference lists of included studies and systematic reviews. We found a total of 2955 articles from the electronic database and one from the university library.

Inclusion Criteria

There was no language restriction. We included studies in English, Spanish, German, randomized control trials (RCT), clinical trials, cross-sectional, case-control, cohort studies, systematic reviews, traditional reviews, opinions, animal studies, letters to the editor editorials. We identified and included studies published in the last 27 years. We had studies conducted in humans of age 10 years onwards.

Exclusion Criteria

Grey literature, books, documents, case series, case reports, overlapping studies, duplicate studies, and studies before 1993.

Results

A total of 2956 studies were obtained by scrutinizing the databases. Records were analyzed based on the title and appropriate abstract and were filtered, applying inclusion-exclusion criteria. We studied a total of 197 reviews that were then filtered. We removed duplicate studies. After setting a 70% benchmark, we assessed 81 studies for quality, and only 41 qualified after applying the quality assessment tools. We used the following means:

Clinical trials = Cochrane Risk Bias Assessment tool, Observational studies= Newcastle Ottawa, AXIS, A systematic review, and meta-analysis = AMSTAR, Animal trials = SYRCLE, Literature review articles = SANRA

Our review includes 367 patients of lichen planus from six different studies, five case-control, and one cross-sectional study depicting the increased prevalence of high homocysteine (HHcy) levels and arterial stiffness. The review includes 621 patients from different studies who benefitted and improved arterial stiffness and elasticity after taking pioglitazone. A total of 17,011 patients from four randomized control trials and a meta-analysis showed reduced inflammation and decreased cardiovascular death. Overall, we found that Pioglitazone pretreatment or early treatment seems promising in mitigating high homocysteine actions.

Discussion

Increased Homocysteine, the Modifiable Culprit: Pathophysiology

**Figure 2:**
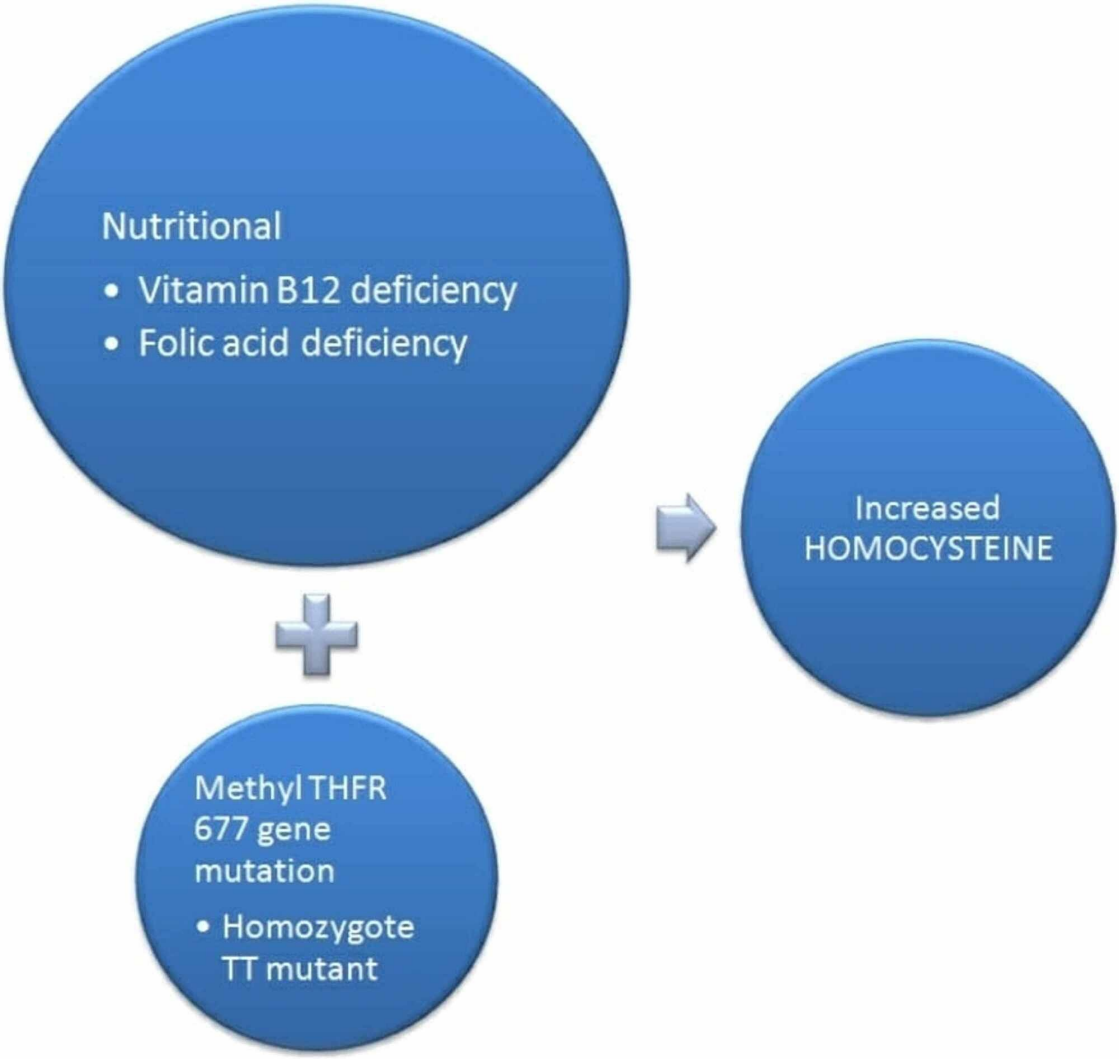
The Diagram Portrays the Mechanisms of Increased Homocysteine Levels in Lichen Planus. MTHFR- methyl tetra hydro folate reductase

Homocysteine, an amino acid part of the methionine pathway, requires both vitamin B12 and folic acid as coenzymes to convert into methionine. A remarkable nutritive inadequacy of folic acid and Vitamin B12 in patients of oral lichen planus (O.L.P.) is seen. As a result, homocysteine levels rise. High homocysteine levels have also been related to disease severity [10]. An analytical study in 2015 revealed abnormally HHcy levels in patients with oral-cutaneous lichen planus and psoriasis [9]. Moreover, certain studies explain the role of the MTHFR 677 gene polymorphism in L.P. [11,12]. The results of Rashed et al. disclosed that folate deficiency and hyperhomocysteinemia were associated with the TT mutant genotype. However, these results were insignificant (P>0.05) [13]. Castro et al. also reported HHcy levels and lower folate levels in homozygoteTT mutants of the MTHFR 677 gene [11]. These results contrast with Kujundzic et al., who said no association between MTHFR genotype and O.L.P. [14]. This inconsistency could be due to the population's alteration as both cutaneous and oral lichen planus patients were studied in the former. In contrast, only patients with oral lichen planus were studied in the latter. The two pathways of increased Homocysteine in L.P., as explained above, are shown below in Figure 2.

**Figure 3:**
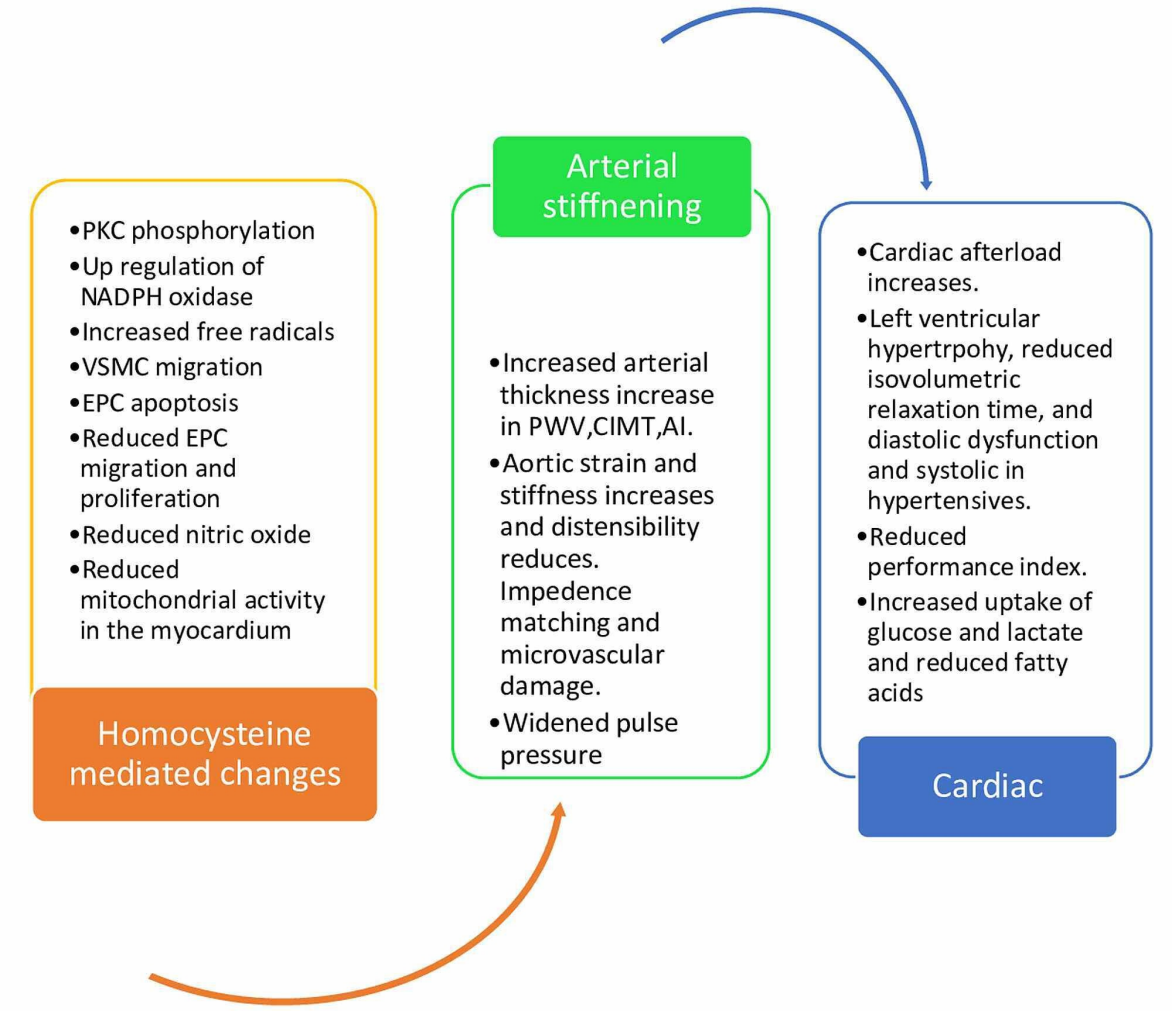
Flowchart Shows High Homocysteine-Mediated Changes in the Cardiovascular System in Lichen Planus. PKC- protein kinase C, NADPH- nicotinamide adenine dinucleotide phosphate, VSMC- vascular smooth muscle cell, EPC- endothelial precursor cells, PWV- pulse wave velocity, CIMT- carotid intima-media thickness, AI- augmentation index

**Figure 4:**
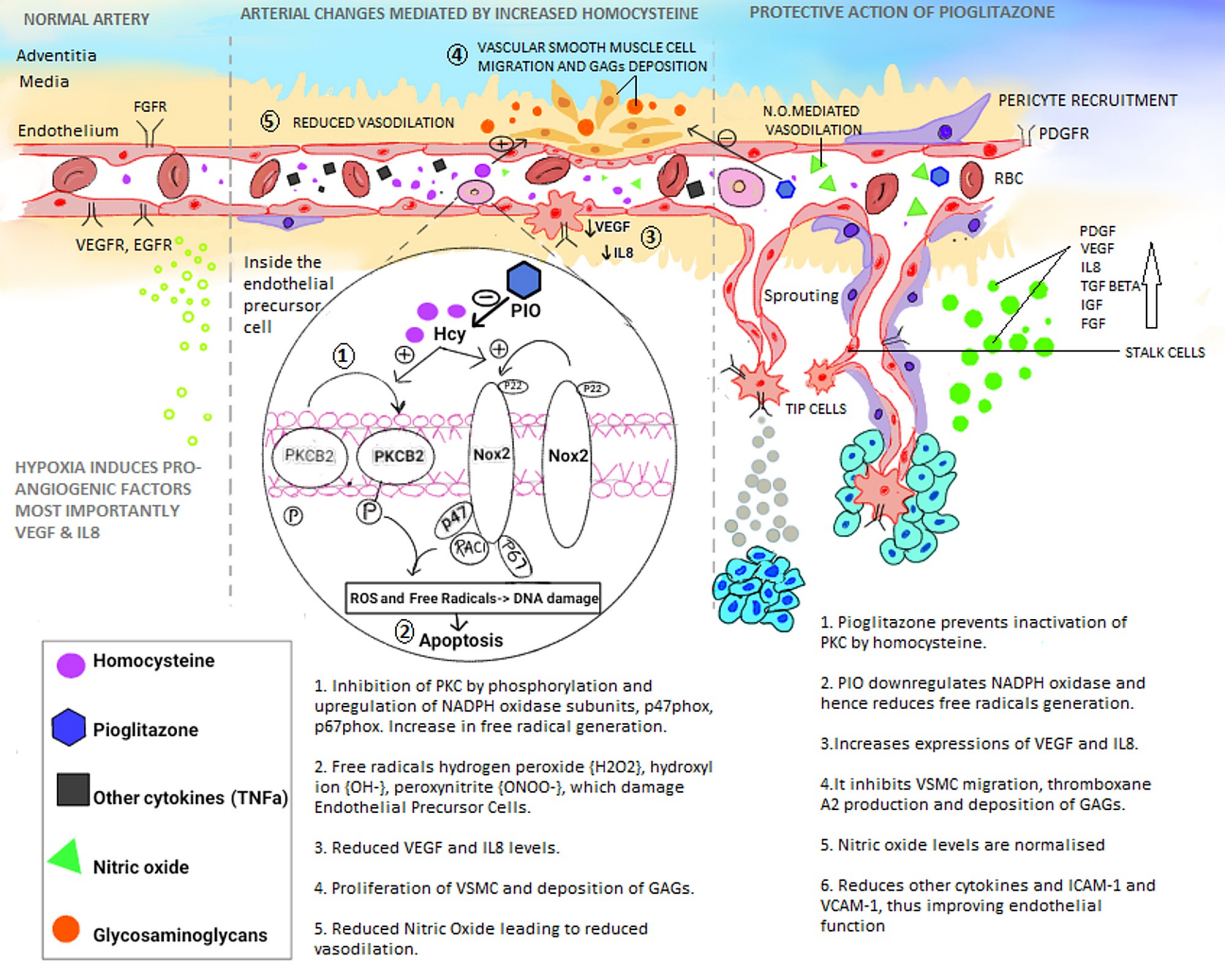
Protective Role of Pioglitazone on Homocysteine Mediated Changes (Left to right: The leftmost column represents the hypoxia-mediated neovascularization in a normal artery. The column in the middle represents the changes mediated by increased homocysteine, and the right column depicts the protective actions of pioglitazone by inhibiting high homocysteine mediated changes. Neovascularization begins with hypoxia and inducible factors. EPC then adheres to the endothelium and migrates towards the hypoxic areas. Migration is stimulated by VEGF, IL8, mainly. Homocysteine mediated ill effects are listed in the middle column, and PIO, overcomes them.) PKC- protein kinase C, NADPH- nicotinamide adenine dinucleotide phosphate, VSMC- vascular smooth muscle cell, EPC- endothelial precursor cells, TNFa- tumor necrosis factor-alpha, GAGs- glycosaminoglycans, PIO- pioglitazone, VEGF- vascular endothelial growth factor, IL8- interleukin 8, TGF Beta- tumor growth factor-beta, FGF- fibroblast-derived growth factor, IGF- insulin-like growth factor, p67phox, p47phox, RAC1 are components of NADPH oxidase, NO- nitric oxide

The possible mechanism behind heightened arterial stiffness due to inflated levels of homocysteine has not been fully comprehended. The central hypothesis suggests increased remodeling of the vascular wall due to magnified oxidative stress [15]. The induced endothelial dysfunction, in turn, encourages the proliferation of vascular smooth muscle cells and deposition of glycosaminoglycans in the matrix, which further impairs vasodilatation [16]. The endothelial cells' growth and integrity may be jeopardized due to reduced methylation potential due to high Homocysteine and S-adenosylhomocysteine levels [17]. During its autooxidation to thiolactone and inhibition of glutathione peroxidase expression, the generation of free radicals has also been suggested (Figures 3, 4) [18]. As shown below, Figure 3 briefly discusses the pathophysiology of high homocysteine-mediated changes in the cardiovascular system.

One of the main mechanisms we would like to emphasize is reducing the number of endothelial precursor cells (EPC) and impairing their functional activities such as adhesion, multiplication, migration, and vasculogenesis capacity. As shown in Figure 3, the mechanism of these effects has been proposed to inhibit protein kinase C (PKC). The manifestation levels of nicotinamide adenine dinucleotide phosphate (NADPH) oxidase subunits, p47phox, NADPH oxidase 2, p67phox, p22phox, RAC-1 (Ras-related C3 botulinum toxin substrate 1) are upregulated by Hcy [19,20]. This results in increased free radicals hydrogen peroxide (H2O2), hydroxyl ions (OH-), peroxynitrite (ONOO-), which damage the endothelium. Furthermore, Hcy induces apoptosis of EPC. Vascular endothelial growth factor (VEGF) and Interleukin 8 (IL8) is suppressed with Hcy [19]. Koller et al. demonstrated that nitric oxide (NO) pathway dysfunction causes reduced dilation of arterioles [20,15]. Augmented production of superoxides has been shown to lessen nitric oxide availability, which modifies the mitochondrial function regulation in the heart. These pathomechanisms also illustrate how HHcy enhances glucose and lactate uptake and curtails free fatty acid uptake by the heart. The concurrently boosted thromboxane A2 (TXA2) activity in the platelets and blood vessels was also narrated. These variations are due to the heightened synthesis of free radicals. They lead to an increase in tumor necrosis factor-alpha (TNFα), NFκbeta (nuclear factor kappa light chain enhancer of B cells), and cause vessels' remodeling (Figures 3, 4) [20]. Eleftheriadou et al. in 2013 demonstrated that acute methionine-induced hyperhomocysteinemia reduces aortic distensibility and worsens the myocardial performance of the left ventricle (Tei index) in healthy individuals [21].

Vascular Remodeling Induced Arterial Stiffness, Effects on the Left Ventricle, and Myocardial Performance Index in Lichen Planus

Vascular stiffness measured in the cardio-ankle vascular index (CAVI) is believed to be a better approach to evaluate arterial stiffness than the conventional method of pulse wave velocity because CAVI is independent of blood pressure [22]. Studies often use carotid-femoral pulse wave velocity (cfPWV), carotid radial PWV, augmentation index (AI), carotid intima-media thickness (CIMT), aortic strain (AS) and distensibility (AD), aortic stiffness index (ASI) to evaluate arterial aging. In 2018, research ascertained that HHcy concentrations were linearly correlated with cfPWV and intensified arterial stiffness [23]. The cf PWV was considerably higher in the high homocysteine group than in the standard. No substantial discrepancy in carotid-radial PWV between the two groups was recognized [15]. A survey by Lim et al. in 2002 concluded that Hcy induced cardiovascular morbidity was slightly reinforced in women compared to men [24]. Sheng et al. in 2015 suggested that homocysteine levels were associated with cfPWV and carotid-ankle pulse wave velocity (caPWV) in men but not in women. This difference may be attributed to the gender difference and faster conversion to Hcy to cystathionine in men [25]. Arterial stiffness has to be described with inflammation, as it may not provide any information alone [26]. Two cross-sectional studies, one in 2018 and the other in 2009, suggested a 14% increase in average arterial stiffness for persistent inflammatory diseases and a positive correlation between cfPWV and years of duration (but not with disease severity), respectively [27,28].

Studies have suggested that high Hcy levels could directly affect the left ventricle and its functions, as illustrated in Figure 3. A survey of 2013 suggests plasma homocysteine levels positively correlate with left ventricular hypertrophy, reduced isovolumetric relaxation time, and diastolic dysfunction [29]. HHcy levels also appear to be involved in left ventricular dilation; however, the resulting hypertrophy is inadequate to reimburse the enhanced wall stress [30]. This may lead to a reduced ejection fraction. Two studies suggested a substantial inverse association of HHcy with LVEF in patients with hypertension, but not in patients with normal blood pressure [31,32]. Rossi et al. also independently confirmed the inverse relationship between LV systolic function and HHcy [31]. High blood pressure enhances the effect of homocysteine levels on arterial stiffness, which is explained by high blood pressure-induced susceptibility of the endothelium to the deleterious impacts of homocysteine. Table 1, shown below, summarizes studies related to the effects of increased homocysteine levels on the left ventricle.

**Table 1:** Effects of Increased Homocysteine Levels on the Left Ventricle. HHcy- high homocysteine, LVM- left ventricle mass, LVH- left ventricular hypertrophy, LVEF- left ventricle ejection fraction, CHD- coronary heart disease, CAD- coronary artery disease, B.P- blood pressure

Study	Author	Year	Type of Study	Patients	Purpose of the study	Results	Conclusion
1.	Kharlamova et al. [29]	2013	Cross-sectional.	109	Impact of HHcy on the left ventricle's morphology and functions in patients on anticipated hemodialysis.	High plasma Hc levels were related to LVM, isovolumetric relaxation time, and E/A ratio.	Plasma homocysteine levels positively correlate with left ventricular (LV) hypertrophy, reduced isovolumetric relaxation time, and diastolic function.
2.	Alter et al. [30]	2010	Cross-sectional.	66	To study the relation of high homocysteine with left ventricular mass and dilatation.	LVM was significantly high, and LV dilatation was more prevalent in hyperhomocysteinemia. Homocysteine does not relate with left ventricular end-diastolic volume, LV end-diastolic wall stress, and LVEF.	High homocysteine appears to be involved in LV dilatation, which is not in proportion to the ensuing hypertrophy. The hypertrophy in turn is inadequate to reimburse for the intensified wall stress.
3.	Rossi et al. [31]	2007	Review		A link between HHcy and left ventricle dysfunction and CHD.	High tHcy indicated cardiovascular mortality in hypertensive, but not in normotensive patients unassisted of coronary heart disease and past record of infarction of heart.	High Hcy was inversely related to left ventricular (LV) ejection fraction. This inverse connection between LV systolic function and high Hcy has been independently verified.
4.	Cesari et al. [32]	2005	Cohort Study.	936	Hyperhomocysteinemia is negatively associated with the ejection fraction of the left heart. It is a good predictable tool for identifying mortality risk factors in patients with CHD and hypertension.	No connection of arterial hypertension or the CAD was found with HHcy. However, a significant inverted relationship of HHcy with reduced LVEF was seen in patients with high B.P	In patients with increased B.P but not in normotensive subjects, HHcy foresees cardiovascular mortality & low ejection fraction, unrelated to CAD or previous episodes of myocardial infarction.

Further, the impact of HHcy may partly be mediated through arterial stiffness. Stiffening of the large vessels, such as that of the aorta, boosts the speed of the pressure wave. The pressure wave that has been reflected reaches the heart faster, at the end of systole rather than the diastole. The systolic blood pressure (SBP) and the cardiac afterload, as a result, increase. This may result in left ventricle remodeling [33]. The left ventricle's wall thickness and mass-volume ratio are negatively associated with the distensibility of the arteries. Arterial stiffness may be associated with LV twisting, reduced myocardial systolic function, and performance index [34]. It produces impedance matching, which reduces wave reflection, exposing the microcirculation to excessive pulsatile stress, resulting in organ damage. 

Our meta-analysis describes the homocysteine-mediated cardiovascular changes in a total of 192 patients of lichen planus from four different case-control studies, as mentioned in Table 2. Baykal et al., in 2020, established an intermediate positive association between length of the disorder and increased arterial stiffness. Impaired systolic and diastolic activities were deduced in patients with lichen planus [35]. Risk is increased in the existence of resistant and chronic disease and with erosive lichen planus. Changes in both the systolic and diastolic functions may be attributed to HHcy's direct effect on the left ventricle and secondary effects due to increased arterial stiffness. Nasiri et al. suggested that patients with L.P. had a significantly greater mean CIMT. A positive relation was also explained between L.P. and HHcy and c-reactive protein (CRP) levels [36]. Koseoglu et al. demonstrated that AS, and AD, were considerably lower, and aortic stiffness index ASI was reasonably elevated in the L.P. group. The myocardial performance index (Tei index) was enormous in this group. They negatively associated L.P.'s duration with the changes and positively associated it with the Tei index and ASIβ [37]. Saleh et al. concluded that L.P. induced greater markers of cardiovascular and metabolic risk factors, probably due to prolonged inflammation. Hence, high Hcy levels and other cytokines may play a role in cardiovascular remodeling and increased morbidity [38]. Below-mentioned, Table 2 depicts the studies included in the discussion concerning the effects of increased arterial stiffness mediated left ventricular changes. Table 3 summarizes the studies discussing the effects of increased homocysteine levels on arterial stiffness in lichen planus and other inflammatory conditions.

**Table 2:** Increased Arterial Stiffness Mediated Left Ventricular Changes. CF PWV- carotid-femoral pulse wave velocity, RWT- relative wall thickness, AI- augmentation index, ESR- erythrocyte sedimentation rate, PP- pulse pressure, hsTNT- high sensitivity troponin T, PWV- pulse wave velocity

Study	Author	Year	Type of Study	Patients	Purpose of the study	Results	Conclusion
1	Zhang et al. [34]	2018	Cross-sectional	147	To assess the association between the structural changes of the left ventricle and arterial stiffness in healthy women.	Cf PWV was significantly related to LV shape and function. RWT apical rotation and LV twisting also seemed related to the arterial stiffness.	It was found that even in healthy women, increased arterial stiffness caused left ventricular twisting. Twisting preceded LV dysfunction.
2	Bai et al. [39]	2011	Cross-Sectional	1479	Arterial stiffness can lead to a minimum elevation in high-sensitivity cardiac troponin T.	High cfPWV and increased PP were associated with a higher likelihood of detectable cardiac troponin T.	A successive subgroup analysis inferred that in patients >60 years, the relation between cfPWV, PP, and hsTnT levels is strengthened.
3	Mitchell [40]	2015	Cohort study	3539	Association between arterial stiffening and cardiovascular events.	Nearly 48 percent of increment was seen in CVS related mortality in patients with higher aortic PWV. A few multivariable models depicted that outcomes of CVS mortality did not depend on AI, PP, and PP amplification.	The higher the aortic stiffness the higher is the risk of developing the first cardiovascular event.
4	O'Rourke and Nichols [33]	2005	Review article		To explain that the diameter of the aorta, its stiffness, and the reflection of pulse waves, increase with age and isolated systolic hypertension		This review concluded that arterial stiffness first leads to a widened pulse pressure and later results in isolated systolic hypertension in the elderly.

**Table 3:** Effects of Homocysteine on Arterial Stiffness in Lichen Planus and Other Inflammatory Conditions. L.P.- lichen planus, CIMT- carotid intima-media thickness, CRP- C reactive protein, MTHFR- methyl tetra hydro folate reductase, O.L.P- oral lichen planus, CYP- cytochrome P, AS- aortic strain, AD- aortic distensibility, ASI- aortic stiffness index, Hcy- homocysteine, HHcy- high homocysteine, cfPWV- carotid-femoral pulse wave velocity, SBP- systolic blood pressure, DBP- diastolic blood pressure

Study	Author	Year	Type of Study	Patients	Purpose of the Study	Results	Conclusions
1.	Baykal et al. [35]	2020	Case-control	55 (L.P.)	To see and correlate vascular stiffness and cardiovascular changes in patients with L.P. against a supervision group.	A reasonable + relation was deduced between the length of disorder and arterial stiffness. Worsening of contraction functions and relaxation functions were also inferred compared to the control group.	A linear relation is seen between the duration of L.P. and arterial stiffness. Cardiovascular risk is enhanced in reluctant and chronic illness, especially in the erosive form of L.P.
2.	Nasiri et al. [36]	2019	Case-control	43 (L.P.)	Examining intima-media thickness of the CIMT and homocysteine statuses in victims of lichen planus.	With the healthy controls, victims of L.P. had a statistically substantial, enormous mean CIMT. A positive relation was also recognized between L.P. and heightened CRP and homocysteine levels.	Measurement of the average CIMT could be advantageous for the timely diagnosis and treatment of atherosclerosis in L.P.
3.	Chen et al. [23]	2018	Cross-sectional study.	16,644	A study was carried out in the adult population to determine the relationship between total homocysteine level and arterial stiffness.	A positive relation was established between HHcy and cfPWV levels and intensified arterial stiffness. Stiffness was much greater in those with Hcy ≥15 umol/L than those with a Hcy level of 10umol/L. A higher prevalence of gained arterial stiffness was established.	The survey ascertained that serum HHcy levels were positively related to cfPWV and enhanced arterial stiffness prevalence. It can be concluded that the cardiovascular consequences of HHcy may somewhat be intervened through arterial stiffness.
4.	Dregan [27]	2018	Cross-sectional	5976	The analysis assessed the assumption that arterial stiffness will increase in inflammatory conditions and specific disorders mediated by inflammation.	Fourteen percent increase in mean arterial stiffness was observed with chronic inflammatory disorders. There was proof for similar associations with other inflammatory diseases, involving psoriasis and rheumatoid arthritis.	It was recorded that the mean arterial stiffness increases with rising quantities of inflammatory biomarkers.
5.	Rashed et al. [13]	2017	Case-control	110 patients (L.P.)	Examining the relation between methylenetetrahydrofolate reductase MTHFR-677 gene polymorphism and CVS danger with L..P.	Patients exhibited a considerably elevated quantity of the MTHFR 677 TT genotype, and its T allele was described in relation to the healthy group.	MTHFR 677 gene polymorphism may be used for developing L.P. and an elevated risk of CVD.
6.	Koseoglu et al. [37]	2016	Case-control	54 (L.P.)	To examine the aorta's elastic properties and the left ventricle's myocardial performance index in L.P.	AS, distensibility of the aorta (AD) were relatively depressed, and the measured aortic stiffness (AS index) was substantially more remarkable. As compared to the healthy control group, the myocardial performance index was higher in patients with L.P.	The L.P. duration was negatively associated with AS and positively correlated with the Tei index and AS Index.
7.	Kujundzic et al. [14]	2015	Cross-sectional	65 (L.P.)	Correlation of MTHFR gene, CYP27b1, and cyp24a1, with the risk of developing oral lichen planus.	No association was found between the MTHFR genotype and O.L.P.	The identification of recent molecular biomarkers could be used in the determination of individuals with O.L.P. propensity.
8.	Saleh et al. [38]	2014	Case-control	40 (L.P.)	To observe the range of CVS related risk factors in L.P.	L.P illustrated a substantial association with metabolic syndrome than controls. Serum HCY, fibrinogen, and high sensitivity C Reactive Protein were considerably inflated in lichen planus.	The current endeavor discovers that L.P. has raised markers of both metabolic syndromes as well as CVS mortality. This has been assumed to be associated with the duration of the illness, as long standing inflammation contributes to the risk factors.
9.	Zang S et al. [15]	2014	Cross-sectional study	1107	To deduce whether there prevails a relation between the concentration of Hcy and arterial stiffness in the aged patients.	The c-fPWV was found to be relatively elevated in the HHcy group than in the conventional group. Although no identifiable change in carotid-radial PWV was found between the two groups.	Increased homocysteine levels strengthen the free radical formation, inflammation of endothelial cells of the blood vessels, and diminish the generation and availability of nitric oxide.
10.	Eleftheriadou et al. [21]	2013	Crossover study	30 healthy volunteers	To understand the effects of acute methionine-induced HHcy on the distensibility of the aorta and PWV Further, to look at the consequences of acute HHcy on the left ventricle's myocardial performance in healthy individuals.	Reduced Aortic distensibility after methionine load and reduced Tei index indicates deterioration.	Acute methionine-induced High HCY diminishes aortic distensibility and worsens myocardial performance in healthy individuals.
11.	Gisondi et al. [28]	2009	Cross-sectional study		To examine if chronic plaque psoriasis is related to enhanced arterial stiffness.	There was a positive correlation between cfPWV and the duration of psoriasis, but not with the severity of the disorder.	Persistent plaque psoriasis may be related to intensified arterial stiffness. The duration of the disease seems more important in atherosclerosis and arteriosclerosis.
12.	Lim et al. [24]	2002	Systematic review			Homocysteine concentration elevation by one standard deviation causes SBP to increase by 0.7mm of Hg and DBP to increase by 0.5mm Hg in males.	Observations showed damage to endothelium-dependent vasodilation and gained oxidative stress in provisional or chronic hyperhomocysteinemia.
13.	Balta et al. [26]	2013	Letter to the editor		Arterial stiffness alone without other characteristics of inflammation may not provide the necessary knowledge to doctors.		Arteriosclerosis, along with inflammatory markers, provides necessary information and evidence to clinicians about the underlying endothelial inflammation.

Protective Role of Pioglitazone: Mechanism of Action

Pioglitazone (PIO), which was introduced as an antidiabetic drug, has now been shown to effectively mitigate the Hcy induced arterial stiffness, improve endothelial function, and decrease cardiovascular morbidity. Arterial stiffness is associated with an increase in cardiac high sensitive Troponin T and increased risk of a first cardiovascular event [38-40]. Zhu et al. in 2018 confirmed that PIO inhibits Hcy- Induced PKC inactivation, as shown in Figure 4, point 1 [19]. Western Blot analysis of EPC treated with PIO demonstrated that it significantly inhibited the phosphorylation of PKC. Flow cytometry studies showed decreased PKC mediated reactive oxygen species intracellularly. The results revealed that the drug attenuates Hcy induced EPC dysfunctions, such as reduced migration and adhesiveness. It also promotes antioxidant properties. PIO also restricts the upregulation of NADPH subunits, Nox2, and p67phox. Further, it has been shown to have pleiotropic effects involving anti-apoptosis and anti-senescence [19]. One of the studies confirmed its effect on VEGF and IL8. As shown in Figure 4, point 3, VEGF AND IL-8, which are otherwise reduced by homocysteine, are normalized by PIO.

PIO's effect is mediated by two intranuclear receptors, the retinoid X receptor, and the PPAR-gamma. A study by Xu et al. in 2017 used GW9662 (5 μM) as a distinctive PPAR-γ blocker to deduce whether PIO exercised its effect on EPCs against Hcy by activating the PPAR-γ receptor [41]. The production of cytokines by EPCs was normalized under the effect of PIO. These results revealed that GW9662 did not prevent PIO from intervening and blocking the Hcy-induced reduction in VEGF and IL-8 production. This suggests that PIO's defensive mechanism occurs via a PPAR-γ-independent pathway [41]. Li et al. illustrated that PIO also blocked Hcy-induced p38 mitogen-activated protein kinase phosphorylation. It inhibited the homocysteine elicited vascular smooth muscle cell migration independent of the PPAR-gamma receptor pathway, as depicted in Figure 4, point 4 [42]. A randomized control trial (RCT) depicted that PIO significantly decreased pulse wave velocity. Besides, it significantly improved aortic elasticity and reduced inflammation [43]. In 2006, another RCT demonstrated a reduction in pro-inflammatory cytokine levels (tumor necrosis factor-alpha, the IL-6, and IL-1 beta) with PIO. Reduced vascular cell adhesion molecule 1 (V-CAM-1) and intercellular adhesion molecule 1 (I-CAM-1) with PIO indicated an improvement in endothelial function. Decreased arterial stiffness was concluded as "AI" was reduced, and PWV decreased by 16.3%. However, significant changes were seen in blood pressure [44]. Another RCT in 2006 with 462 patients concluded after a one and a half year treatment period that pioglitazone delayed the advancement of CIMT [45]. The drug improves endothelial vasomotion, inhibits procoagulant processes, and has powerful antiproliferative and antioxidant effects [46]. Methylation of enzymes generally silences them. PIO reduces inducible nitric oxide synthase (iNOS) DNA methylation by downregulating the process and thus increases the activity of iNOS [47]. This has been represented in Figure 4, point 5. Overall, PIO modestly lowers blood pressure, reduces microalbuminuria, arterial stiffness, and reduces carotid wall thickening. It reduces the effects on the heart and decreases cardiac remodeling. Figure 4 depicting the mechanism of action of PIO against HHcy mediated arterial stiffness.

A meta-analysis involving 16,390 patients [48] and the PROactive (PROspective pioglitAzone Clinical Trial In Macrovascular Events) trial of PIO concluded a reduction in cardiovascular mortality and non-fatal myocardial infarction. Although hospitalization rates due to heart failure inflated significantly, the death rates for heart failure did not change [49]. The insulin resistance intervention after stroke (IRIS) trial that analyzed the effect of pioglitazone on future cardiovascular events also had comparable results. Some critics assert that the results showed a significant decline in cardiovascular consequences. Others debated the design and statistical analyses, and they evaluated the deductions to be flawed [49]. One discovery that everybody acknowledges is that PIO increases hospitalization incidence but not the mortality for congestive heart failure. Its impact on the heart remains inconclusive, and it is contraindicated in patients with New York Health Association Functional Classification (NYHA) class III or IV. Warnings exist for its use in any patient with heart failure [50]. Research has also shown an increased risk of fractures and weight gain as its most common side effects in high-risk patients. As shown below, Table 4 includes studies describing the protective role of pioglitazone by blocking homocysteine mediated arterial changes.

 

**Table 4:** Studies Describing Pioglitazone's Protective Role by Blocking Homocysteine-Mediated Arterial Changes and the Potential Complications of The Medication. EPC- endothelial precursor cells, PKC- protein kinase C, VEGF- vascular endothelial growth factor, NADPH oxidase- nicotinamide adenine dinucleotide phosphate, PPAR-gamma- peroxisome proliferator-activated receptors, MAPK- mitogen-activated protein kinase, DM- diabetes mellitus, RCT- randomized control trial, iNOS- inducible nitric oxide synthase, ICAM 1- intercellular adhesion molecule 1, VCAM 1- vascular cell adhesion protein 1, PWV- pulse wave velocity, AI- augmentation index, CAD- coronary artery disease, PIO- pioglitazone, RA- rheumatoid arthritis

Study	Author	Year	Type of Study	Patients	Purpose of the Study	Results	Conclusions
1	Zhu et al. [19]	2018	Molecular study/clinical experiment	Eight healthy volunteers.	Mechanism of action of PIO in protecting against and preventing Hcy induced EPC changes.	PIO reduces the expressions of Nox2 and p67phox.	PIO achieves restoration of the EPCs by inactivating protein kinase C and blocking NADPH oxidase.
2	Xu et al. [41]	2017	Animal study	Peripheral blood mononuclear cells were taken from healthy volunteers.	PKC/NADPH oxidase is blocked in pioglitazone's protective mechanism against high homocysteine-induced paracrine dysfunction in endothelial progenitor cells.	The result indicated that the defensive effect of PIO in normalizing Hcy-induced reduction in VEGF and IL-8 occurs via a PPAR-γ-independent pathway.	Production of inflammatory cytokines by EPCs (stimulated with Hcy) was brought back to normal levels by PIO. PIO intervenes via a PPAR-gamma independent pathway.
3	Marder et al. [43]	2013	RCT	143 (outcomes diabetic RA pts)		Pioglitazone significantly decreased pulse wave velocity.	The inclusion of pioglitazone in the routine management regime of rheumatoid arthritis considerably improved the elasticity of the aorta, along with decreasing inflammation.
4	Li et al. [42]	2008	Molecular study/clinical experiment		PIO blocks Hcy-induced vascular smooth muscle cells migration by a pathway other than the PPAR-gamma receptor-mediated.	Not only does PIO inhibit NADPH oxidase, but it also blocks Hcy induced p38 MAPK phosphorylation.	Pioglitazone also mediates its action by blocking Hcy-induced MAPK phosphorylation.
5	Rohatgi et al. [50]	2008	Review		To study the consequences and complications of thiazolidinediones on microvasculature- and macro vasculature in patients with diabetes mellitus.	A ten percent reduction in mortality related to CAD and peripheral vascular disease and revascularization was seen with PIO.	Use of this medication is threatened in patients with a history of heart failure NYHA class III or IV, hence contraindicated in these patients.
6	Lincoff et al. [48]	2007	Meta-analysis	16,390	To assess the effects of pioglitazone therapy on ischemic cardiac episodes.	The beneficial impact of pioglitazone on ischemia-related episodes and heart failure was the same across trials of varying lengths, comparators, and patients with or without vessel-related diseases.	PIO is known to be related to a lower risk of myocardial infarction and stroke among patients with diabetes mellitus. Incidence of heart failure is increased by PIO, although associated mortality is not affected.
7	Jiang et al. [47]	2007	Molecular study		PIO's effects on iNOS in the presence of Hcy in human monocytes.	Hcy decreases the expression of iNOS-by-iNOS DNA methylation, reducing transcription. PIO can downregulate iNOS-DNA- methylation and increase the activity of iNOS, thus restoring levels of nitric oxide.	Pioglitazone inhibits homocysteine-induced DNA methylation of inducible nitric oxide synthase genes.
8	Goldberg [46]	2007	Review		Thiazolidinedione and vascular damage.	The drug improves endothelial vasomotion, inhibits inflammatory and procoagulant activities. It has strong antiproliferative and antioxidant actions.	They lower blood pressure, microalbuminuria, arterial stiffness, and CIMT. These effects are independent of glucose-lowering and have been shown to occur in nondiabetic subjects too.
9	Ryan et al. [44]	2006	RCT	16 (on pioglitazone)	Endothelial function improves, and arterial stiffness reduces obese glucose-tolerant men when treated with fenofibrate and pioglitazone.	A decrease in ICAM 1 and VCAM1 indicates an improvement in endothelial function with both pioglitazone and fenofibrate. Reduced arterial stiffness was concluded as PWV and AI were reduced. Blood pressures did not decline significantly.	It was concluded that inflammation, adhesion molecules, and arterial stiffness were reduced by fenofibrate and PIO. Long-term use of these in the prevention of CAD has been suggested.
10	Mazzone et al. [45]	2006	RCT	462	To evaluate the effect of PIO versus glimepiride on CIMT in patients with type 2 diabetes mellitus.	PIO decelerates the progression of CIMT more than glimepiride. The effect of pioglitazone was similar across many subgroups.	Over a one-and-a-half-year treatment period, patients with type 2 DM benefited with PIO more than with glimepiride, and CIMT reduced more with PIO.

Limitations

**Figure 5:**
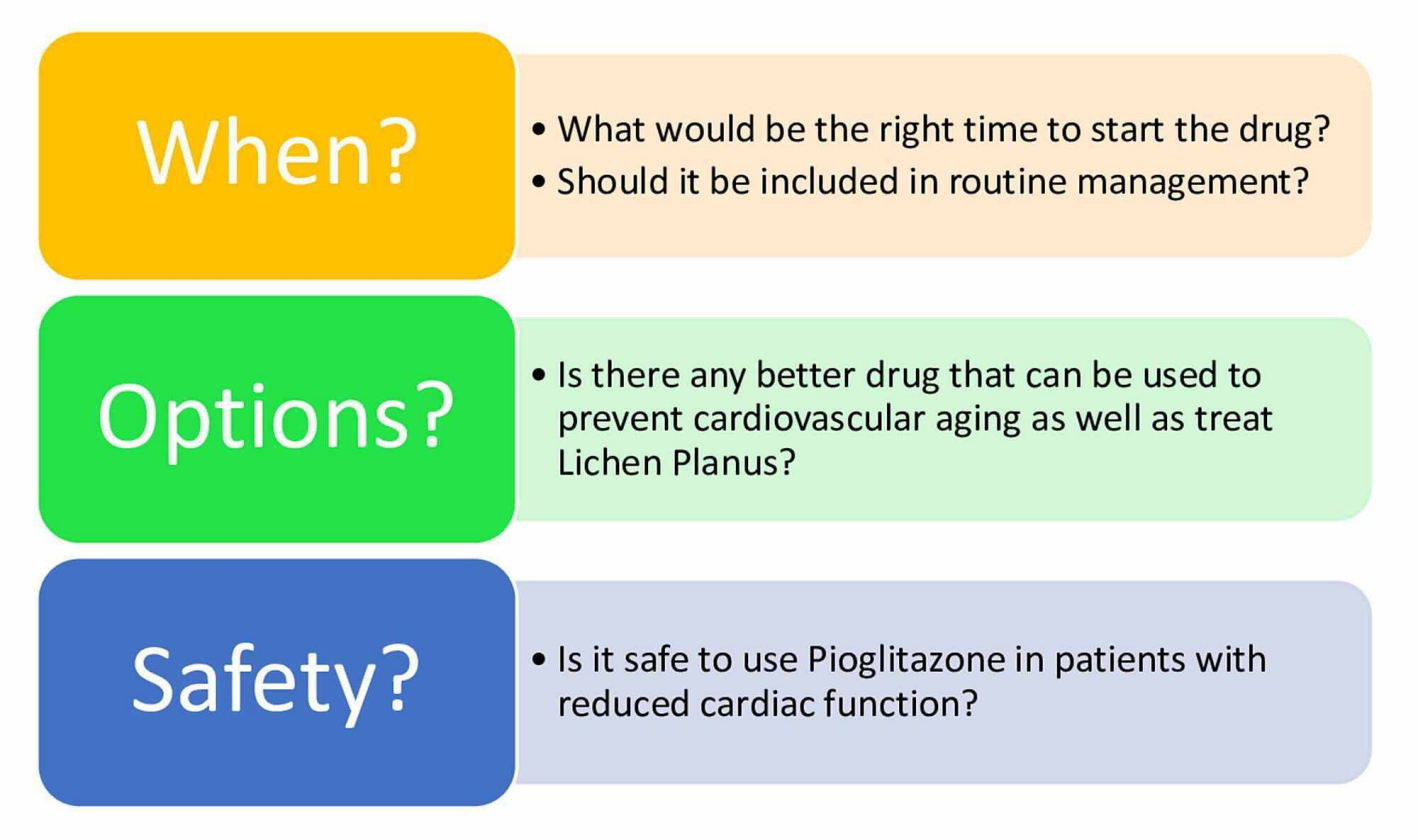
Box Describing Questions That Need to Be Explored Further.

PIO seems to have promising effects in alleviating the Hcy induced arterial changes and cardiac remodeling; however, its safety profile on the heart remains uncertain. Many authors seem to have conflicting remarks about the adverse effects of the drug. Moreover, we found no RCTs or case-control studies where patients with L.P. were treated with PIO. PIO has been used previously in patients with Lichen Planus. However, its effectiveness in the other subtypes is less known. Our systematic review will hopefully pave the way for more exploration of this topic. The box (Figure 5), as shown below, shows questions that remain unanswered and need further investigation.

## Conclusions

We aimed to assess if pioglitazone is the new generation drug against homocysteine induced changes in L.P and whether it can be used in routine management. We deduced that nutritional deficiency, genetic polymorphism, and disease severity of L.P increase homocysteine. High Hcy leads to intima-media damage and apoptosis of EPCs thus reducing neovascularization. It also decreases nitric oxide-mediated vasodilation. It further increases free radicals, VSMC proliferation, and GAG deposition. HHcy lowers the mitochondrial quantity in the heart; increases afterload leading to LV hypertrophy, dilation, & diastolic dysfunction. Thus, reducing myocardial performance overall. Impedance matching damages microcirculation.

PIO, an anti-apoptotic, enhances neovascularization, vasodilation and reverts all the above mechanisms induced by HHCY, in ways independent of its PPAR- gamma receptor pathway. Cardiac changes and incidence of fatal cardiac events are also reduced. Surveys claim it to be used for specific subtypes of lichen planus. Assessing the risk-benefit ratio, we should give PIO a fair trial in managing patients with lichen planus with no history of cardiac disease. Given early, it might prevent cardiovascular and inflammation-related morbidity. Our research on this topic is crucial as these underlying processes, which are otherwise preventable, are often overlooked and intensify patient morbidity. Questions like when should the drug be initiated and in whom and whether it is safe, or is there a better drug to treat L.P. and mitigate the ill effects HHcy concurrently, remain unanswered. Our survey will hopefully pave the way for more exploration.
